# Supervised PolSAR Image Classification with Multiple Features and Locally Linear Embedding

**DOI:** 10.3390/s18093054

**Published:** 2018-09-12

**Authors:** Qiang Zhang, Xinli Wei, Deliang Xiang, Mengqing Sun

**Affiliations:** 1 School of Chemical Engineering and Energy, Zhengzhou University, Zhengzhou 450001, China; ZQ_28@126.com; 2 Institute of Surveying and Mapping, Information Engineering University, Zhengzhou 450001, China; 3 National Innovation Institute of Technology, Beijing 100071, China; 4 College of Surveying and Geo-Informatics, North China University of Water Resources and Electric Power, Zhengzhou 450011, China; mqsun@126.com

**Keywords:** land-cover classification, superpixel-based, multiple-component decomposition, supervised locally linear embedding (S-LLE), polarimetric synthetic aperture radar (PolSAR)

## Abstract

In this paper, we propose a new method of land use and land cover classification for polarimetric SAR data. This algorithm consists of three parts. First, the multiple-component model-based scattering decomposition technique is improved and the decomposed scattering powers can be used to support the classification of PolSAR data. With this decomposition, the volume scattering of vegetated areas is enhanced while their double-bounce scattering is reduced. Furthermore, the double-bounce scattering of urban areas is enhanced and their volume scattering is decreased, which leads to an improvement in the classification accuracy especially for the urban areas. Second, this classification strategy is carried out on the superpixel level, which can decrease the influence of speckle noise and speed up the classification. Moreover, the contexture and spatial features extracted from these superpixels are utilized to improve classification accuracy. Lastly, we introduce the supervised locally linear embedding approach to map the high dimensional features into the low dimensional features as the inputs of classifiers. The classification is completed using the nearest neighbor classifier. The effectiveness of our proposed method is demonstrated using the AIRSAR C-band PolSAR data set, which is compared with the original MCSM-SVM and newly published LE-IF PolSAR classification methods. Further investigation is also carried out on the individual contribution of the three parts to LULC classification using AIRSAR C-band data. It indicates that all three components have important contributions to the final classification result.

## 1. Introduction

Polarimetric synthetic aperture radar (PolSAR) can provide more useful information of targets with four polarizations than the single polarization SAR. Therefore, the PolSAR data has been used for various remote sensing applications such as land cover classification, urban extraction, and analysis [1,2,3]. Land cover classification has attracted more and more attention. However, due to the speckle noise within the PolSAR data, the image classification is still a challenge. Until now, many supervised and unsupervised PolSAR image classification methods have been proposed to resolve this issue [4,5,6,7,8,9]. In these two kinds of classification strategies, feature selection is the key element since a set of suitable features may get a correct classification even if using a simple classifier. In contrast, it could be difficult to achieve the satisfactory land cover classification without well-selected features even if using a complex and advanced classifier [10]. The features extracted from the PolSAR image include the physical scattering features obtained from the various decomposition methods and the statistical contexture features. Some polarimetric decomposition theorems have been introduced [11,12,13,14,15] and classification methods based on decomposition results have been explored [7,8,9,16,17]. However, there are some misclassifications in this kind of scattering-mechanism-based PolSAR land cover classification for the reason that some different classes may have the same scattering mechanism and the same classes can exhibit different scattering mechanisms especially for the oriented urban areas and the vegetation [2,18,19]. To resolve this issue, a wide variety of polarimetric features are used including the decomposition powers and several polarimetric indexes such as backscattering coefficients of different polarizations (linear: HH, HV, VV; circular: LL, RR, RL; and linear 45°, 45C, 45X) and their ratios. In addition to polarimetric information, some studies on PolSAR image classification are also researched from the prospects of image understanding, which indicate the effectiveness of image texture descriptors on classification [20]. Recently, some studies have indicated that the fusion of physical and textural information derived from various SAR polarizations is helpful in improving classification results. Tu et al. [10] proposed the combination of various decomposition scattering powers, backscattering coefficients, and phase differences between co-polarizations and cross-polarizations as well as some other polarimetric signatures for PolSAR image classification. Qi et al. [21] utilized the decomposition scattering powers, image texture, and the interferometry information to achieve classification for RADARSAT-2 data. Zhang et al. [22] utilized the scattering powers and GLCM texture features for the ESAR image classification. While these integration methods can make full use of image information and significantly improve classification accuracy, some deficiencies still exist. First of all, various features have information redundancies. For instance, the Krogager rotation angle is relative to the polarization orientation angles and the H/alpha parameters describe the chaotic volume scattering, which is also considered in the Freeman–Durden methods. These information redundancies may lead to low classification accuracy. Even though some dimensionality reduction techniques are utilized to diminish these redundancies [10,23,24], the computation time of these methods for so many features are very large, which makes the classification techniques uneasy to use. Second, these classification methods are mostly pixel-based, which results in the sensitivity to speckle noise and large computation load.

Considering the decomposition drawbacks and features redundancies in this paper, we propose an improved multiple-component model-based decomposition method for the sake of PolSAR image classification. It consists of two main improvements. First, the reorientation process is applied to the coherency matrix before it is decomposed into five scattering components. Then, two suitable volume scattering models designed for forests and oriented urban buildings are used in the decomposition. The advantage of the proposed decomposition is that volume scattering of vegetation is enhanced while its double-bounce scattering is reduced. Moreover, double-bounce scattering of urban buildings is enhanced and its volume scattering decreases. Therefore, the scattering powers obtained using the improved decomposition method has a good ability in discriminating the urban areas. After that, five decomposition powers are selected for the classification instead of using numerous polarimetric features.

Compared with pixel-based image classification methods, region-based classification is a promising scheme. After segmenting images under some constraints such as intensity, location, texture, and edge, we can get many homogeneous regions and then classification is based on these regions instead of pixels. A superpixel [25] denotes a local, coherent region, which is approximately homogeneous in size and shape just like pixels. Xiang et al. [25] proposed a superpixel generating algorithm based on pixel intensity and location similarity for the SAR image and extracted the Gabor filters and GLCM from each superpixel for classification. For PolSAR data, Xiang et al. [26] proposed an adaptive superpixel generation method based on the spherically invariant random vector product model, which can generate satisfactory superpixels with good boundary adherence and compactness. In this paper, we extract the superpixels using Xiang’s method [26]. Afterwards, GLCM features and the spatial relationships (e.g., mean and variance values of each superpixel) are extracted for superpixel-based classification.

Even though the number of polarimetric features is much less than those of other classification methods, texture features and spatial information also have some feature redundancies. There are many linear and nonlinear dimensionality reduction methods proposed to project high-dimensional data into a new space of lower dimensionality including principal component analysis (PCA) [27], linear discriminated analysis (LDA) [28], locally linear embedding (LLE) [29], isometric feature mapping (ISOMAP) [30], and Laplacian eigenmaps (LE) [31]. Shi et al. [24] used a linear dimensionality reduction technology named SGE to obtain a low-dimensional subspace that can preserve the discriminative information from training samples. Tu et al. [10] pointed out that it is more effective to use a nonlinear local dimensionality reduction method considering the nonlinearity of the polarimetric manifold. Hence, they used Laplacian eigenmaps to map the high dimensional polarimetric features into lower dimensionality feature vector for PolSAR image classification. However, this method is an unsupervised learning algorithm, which means it assumes no prior information on the input data. In addition, the size of the neighborhood needs to be set before mapping, which is inflexible [32]. To improve the classification performance, the discriminative information from the given training samples should be considered. Considering the advantages of LLE such as being non-iterative and avoiding the local minima problems in this paper, we propose to use the supervised locally linear embedding (S-LLE) to reduce the feature redundancies. It has favorable properties. (i) It adaptively estimates the local neighborhood surrounding each sample and (ii) the objective function simultaneously maximizes the local margin between heterogeneous samples and it also pushes the homogeneous samples closer to each other.

The main contributions of this paper mainly lie on the following aspects: (1) the improved decomposition scattering powers proposed for PolSAR image classification, (2) superpixel-based classification strategy, and (3) supervised locally linear embedding approach for feature dimensionality reduction. Even though the superpixel-based classification strategy is already widely used, the scattering power features extracted from the superpixels and the dimensionality reduction are both improvements we proposed in this work. Therefore, the contributions and structure of our method are dramatically different from the existing approaches. The remainder of this paper is organized as follows. Section 2 describes the decomposition scattering powers obtained from the improved multiple-component decomposition technique. In Section 3, the superpixels generated from PolSAR data and the corresponding features extraction are described. In Section 4, the S-LLE technique is described and the dimensional reduction performances of different methods are compared. We show the study area and further compare the experimental results with other methods in Section 5. Section 6 concludes the paper.

## 2. Decomposition Scattering Powers

In this study, we depict the improved multiple-component decomposition algorithm, which includes the reorientation processing of the polarimetric coherence matrix, two volume scattering models designed for the vegetation and oriented buildings, and the branch condition to decide which land cover contributes to the volume scattering.

### 2.1. Deorientation Processing of the Coherence Matrix

The fully polarimetric SAR system obtains a 2×2 scattering matrix S for each pixel with four polarizations HH, HV, VH, and VV like [33].

(1) $$S=\left[\begin{array}{cc} S_{\mathrm{HH}} & S_{\mathrm{HV}}\\ S_{\mathrm{VH}} & S_{\mathrm{VV}} \end{array}\right]$$

where $S_{\mathrm{HH}}$, $S_{\mathrm{HV}}$, $S_{\mathrm{VH}},$ and $S_{\mathrm{VV}}$ represent the complex scattering coefficients. Then the Pauli vector $k_{p}$ is defined below [33].

(2) $$k_{p}=\frac{1}{\sqrt{2}}{\left[S_{\mathrm{HH}}+S_{\mathrm{VV}},S_{\mathrm{HH}}-S_{\mathrm{VV}},2S_{\mathrm{HV}}\right]}^{T}$$

which assumes the reciprocal condition of $S_{\mathrm{HV}}=S_{\mathrm{VH}}$. The corresponding coherency matrix 〈[T]〉 can be created from the Pauli vector $k_{p}$ and defined by the formula below.

(3) $$\langle [T]\rangle =k_{p}k_{p}^{†}=\left[\begin{array}{ccc} T_{11} & T_{12} & T_{13}\\ T_{12}^{*} & T_{22} & T_{23}\\ T_{13}^{*} & T_{23}^{*} & T_{33} \end{array}\right]$$

where the symbol 〈·〉 represents the ensemble average and the superscript † denotes the complex conjugation and transposition. To minimize the cross-polarization term $T_{33}$, the coherency matrix 〈[T]〉 is generally rotated by an angle θ as seen in the equation below.

(4) $$\left[T^{\prime }\right]=\left[R\left(\theta \right)\right]\left[T\right]{\left[R\left(\theta \right)\right]}^{†}$$

where [R(θ)] is the rotation matrix and is depicted by the formula below.

(5) $$\left[R\left(\theta \right)\right]=\left[\begin{array}{ccc} 1 & 0 & 0\\ 0 & \cos2\theta & \sin2\theta \\ 0 & -\sin2\theta & \cos2\theta \end{array}\right]$$

Then, the rotation angle can be obtained by making the derivative of $T_{33}^{\prime }$ with respect to θ equal to zero. Therefore, the expression for the rotation angle is represented by Equation (6) below [34].

(6) θ=14tan−1(2Re{T23}T22−T33)

where Re{T23} is the real part of $T_{23}$. In this way, the cross-polarization term $T_{33}$ is minimized and the other terms of the rotated coherency can be obtained according to Equations (3) and (5).

**Figure 1:**
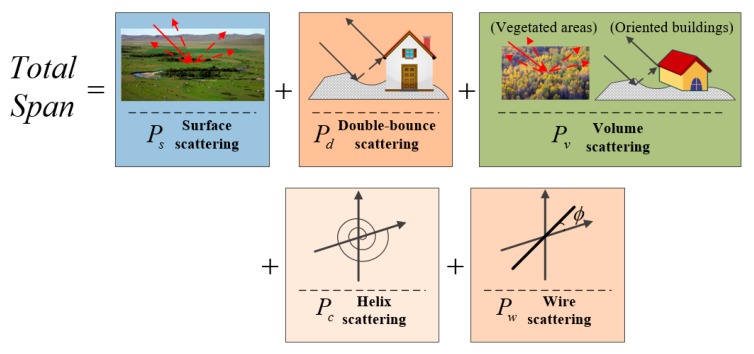
Improved multiple-component model-based decomposition framework.

After the rotation, the measured coherency matrix can be decomposed into five components similar to the original multiple-component model-based (MCSM) decomposition [12], which is shown in Figure 1. These correspond to the surface scattering, double-bounce scattering, volume scattering, helix scattering, and wire scattering mechanisms [12]

(7) $$\langle [T]\rangle =f_{s}{\langle [T]\rangle }_{\mathrm{surface}}+f_{d}{\langle [T]\rangle }_{\mathrm{double}}+f_{v}{\langle [T]\rangle }_{\mathrm{volume}}+f_{h}{\langle [T]\rangle }_{\mathrm{helix}}+f_{w}{\langle [T]\rangle }_{\mathrm{wire}}$$

where $f_{s},\;f_{d},\;f_{v},\;f_{h},$ and $f_{w}$ are the expansion coefficients to be determined. ${\langle [T]\rangle }_{\mathrm{surface}}$, ${\langle [T]\rangle }_{\mathrm{double}}$, ${\langle [T]\rangle }_{\mathrm{volume}}$, ${\langle [T]\rangle }_{\mathrm{helix}},$ and ${\langle [T]\rangle }_{\mathrm{wire}}$ are expansion matrices corresponding to the surface, double-bounce, volume, helix, and wire scattering mechanisms, respectively.

The expansion matrices for the surface scattering, double-bounce scattering, helix scattering, and wire scattering in Equation (6) are the same as those in the original MCSM decomposition, which can be found in Reference [12]. The only difference is the volume scattering models, which are designed for vegetated areas and the orientated buildings, respectively. In the MCSM decomposition, the volume scattering model ${\langle [T]\rangle }_{\mathrm{volume}}$ is defined by the formula below.

(8) $${\langle [T]\rangle }_{\mathrm{volume}}=\frac{1}{4}\left[\begin{array}{ccc} 2 & 0 & 0\\ 0 & 1 & 0\\ 0 & 0 & 1 \end{array}\right]$$

which is the same as that in the Yamaguchi four-component model-based decomposition [11,13].

Generally, buildings parallel to the radar flight path have strong double-bounce scattering and can be modeled by the dihedral corner reflector with a zero polarization orientation angle [13,15]. Nevertheless, for buildings not parallel to the radar flight path, i.e., the orientated buildings in Figure 1, the cross-polarized component is generated, which exhibits a relatively large term in the scattering matrix [26,35,36,37]. This cross-polarized component of the oriented buildings contributes to the HV scattering component dramatically. Since the original volume scattering of MCSM, as depicted in Equation (8) assumed that HV component solely reflects the volume scattering, there will be overestimation of volume scattering in the orientated buildings. To resolve this problem, the volume scattering model is modified by many methods to reduce the volume scattering and enhance double-bounce scattering for the urban areas at the same time [38,39,40,41]. Nevertheless, these new volume scattering models may bring underestimation of volume scattering over vegetated areas [42]. Consequently, in this paper, we propose to use two different volume scattering models to describe the HV scattering of vegetated areas and orientated buildings, which will be presented in the following subsection.

### 2.2. Two Different Volume Scattering Matrices

It is demonstrated that both surface scattering and double-bounce scattering are definite scatterings while the volume scattering is not. The volume scattering represents a chaotic scattering state and it can be regarded as a combination of several kinds of scattering mechanisms [41]. The cross-polarization term, i.e., $T_{33}$, is induced both by vegetated areas and oriented buildings. For these two land covers, there should be two different matrices to describe the scattering.

As we know, the volume scattering model proposed by Freeman assumes that volume scatterers consist of a cloud of randomly oriented dipoles and the orientation angle distribution is uniform. In fact, the elementary volume scatterers can be extended from dipoles to more general targets with the following scattering matrix when in standard orientation [15].

(9) $$S=\left[\begin{array}{cc} S_{\mathrm{HH}} & 0\\ 0 & S_{\mathrm{VV}} \end{array}\right]$$

**Figure 2:**
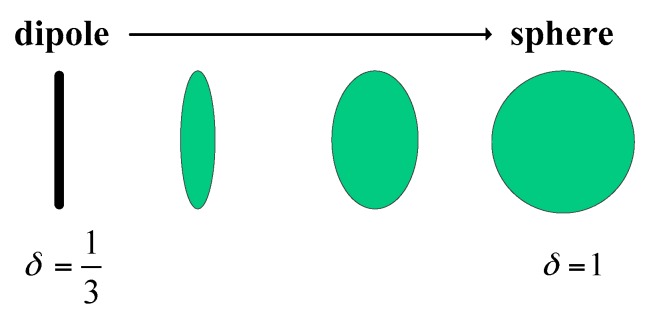
Varying the shape parameter δ.

The orientation angles of vegetation are still assumed to respect the uniform distribution. Then the incoherent averaging of these targets is given by Equation (10) below [14,33].

(10) $${\langle [T]\rangle }_{\mathrm{volume}}^{\mathrm{veg}}=\int_{-\pi }^{\pi }\frac{1}{2\pi }T_{r}\left(\theta \right)d\theta$$

where $T_{r}\left(\theta \right)$ denotes the coherency matrix of the elementary scatterer. Using Huynen’s parameters, we can get the integration result as shown by the formula below.

(11) $${\langle [T]\rangle }_{\mathrm{volume}}^{\mathrm{veg}}=\left[\begin{array}{ccc} 2A_{0} & 0 & 0\\ 0 & B_{0} & 0\\ 0 & 0 & B_{0} \end{array}\right]$$

where $A_{0}=\frac{1}{4}{\left|S_{\mathrm{HH}}+S_{\mathrm{VV}}\right|}^{2}$ and $B_{0}=\frac{1}{4}{\left|S_{\mathrm{HH}}-S_{\mathrm{VV}}\right|}^{2}$. If we use a parameter δ to describe the relationship between $A_{0}$ and $B_{0}$, then Equation (11) is depicted below.

(12) $${\langle [T]\rangle }_{\mathrm{volume}}^{\mathrm{veg}}=\frac{1}{3-\delta }\left[\begin{array}{ccc} 1+\delta & 0 & 0\\ 0 & 1-\delta & 0\\ 0 & 0 & 1-\delta \end{array}\right]$$

where $\left(2A_{0}/B_{0}\right)=\left(1+\delta \right)/\left(1-\delta \right)$ and the normalization coefficient 1/(3−δ) is used to make the trace equal to one. Actually, Equation (12) is the same as the volume scattering model used in References [43,44] and is effective in the case of volume scattering from vegetated areas. The difference is the physical meaning of δ. Freeman pointed out that the volume component can be modified by varying the shape parameter δ that runs continuously from δ=1/3 (corresponding to dipoles) to δ=1 (corresponding to spheres), which is shown in Figure 2.

However, if we consider that δ=0, we can get the volume scattering model shown in the equation below.

(13) $${\langle [T]\rangle }_{\mathrm{volume}}^{\mathrm{veg}}=\frac{1}{3}\left[\begin{array}{ccc} 1 & 0 & 0\\ 0 & 1 & 0\\ 0 & 0 & 1 \end{array}\right]$$

where it can be observed that this model is also a cloud of elementary scatterers as that of the Freeman volume scattering model. The difference is the value of δ. When δ=1/3, we get Freeman volume scattering as depicted in Equation (8), which can be decomposed as seen in Reference [41].

(14) $${\langle [T]\rangle }_{\mathrm{volume}}=\frac{3}{4}{\langle [T]\rangle }_{\mathrm{volume}}^{\mathrm{veg}}+\frac{1}{4}\left[\begin{array}{ccc} 1 & 0 & 0\\ 0 & 0 & 0\\ 0 & 0 & 0 \end{array}\right]$$

It can be observed that the Freeman volume scattering model regards some surface scattering as volume scattering. If δ=1, Equation (12) becomes $\left[\begin{array}{ccc} 1 & 0 & 0\\ 0 & 0 & 0\\ 0 & 0 & 0 \end{array}\right]$, which is a pure surface scattering model. Therefore, we can conclude that if δ>0, more surface scatterings are regarded as volume scattering caused by vegetation. In contrast, if δ=−1, Equation (12) becomes $\frac{1}{2}\left[\begin{array}{ccc} 0 & 0 & 0\\ 0 & 1 & 0\\ 0 & 0 & 1 \end{array}\right]$ where the elementary volume scatterers are small dihedrals. This kind of volume scattering model only consists of a cloud of double-bounce scatterers. Hence if δ<0, there are more double-bounce scatterings in the volume scatting model. So parameter δ can be regarded as a trade-off factor. From the above analysis, we can conclude that δ=0 corresponds to pure volume scattering caused by vegetation, which is shown in Equation (13).

For oriented buildings, since the buildings usually have different orientation angles, which will influence the building scattering mechanism significantly, we introduce the cross scattering model to describe the volume scattering of buildings, which was proposed by Xiang et al. in [45]. This cross scattering model incorporates the dominant orientation angle of buildings into the cosine distribution to implement ensemble averaging of dihedral corner reflectors, which makes it flexible and adaptive in the model-based decomposition. The volume scattering model for oriented buildings is defined by Equation (15) below [45].

(15) $${\langle [T]\rangle }_{\mathrm{volume}}^{\mathrm{ori}}=\left[\begin{array}{ccc} 0 & 0 & 0\\ 0 & \frac{1}{2}-\frac{1}{30}\cos\left(4\theta_{\mathrm{dom}}\right) & 0\\ 0 & 0 & \frac{1}{2}+\frac{1}{30}\cos\left(4\theta_{\mathrm{dom}}\right) \end{array}\right]$$

where the dominant angle of buildings $\theta_{\mathrm{dom}}$ can be obtained using Equation (6) in a local image patch. Further information about the cross scattering model can be found in Reference [45].

### 2.3. Branch Condition

Since we considered to use two different volume scattering models for the vegetated areas and oriented buildings respectively, there should be a branch condition to determine whether the volume scattering is induced by vegetation or by the oriented buildings. It is already known in References [11,13,46] that the double-bounce scattering caused by the oriented buildings makes Re{SHHSVV∗}<0 while a non-dihedral structure such as the vegetated area leads to Re{SHHSVV∗}>0. Therefore, similar to Reference [46], we get the following equation for the Re{SHHSVV∗} as shown in Equation (16) below.

(16) Re{fsβ+fdα∗}+13fv−14fc+Re{γ}fw=Re{SHHSVV∗}

where β, α, and γ are the parameters involved in the scattering matrices of surface, double-bounce and wire scattering mechanisms [12]. Based on this equation, we can get the condition in the formula shown below.

(17) C1=T11−T22+12fc−2Re{γ}fw.

Therefore, if $C_{1}>0$, it can be decided that the volume scattering is caused by vegetated areas while, if $C_{1}\leq 0$, the volume scattering is deduced by the oriented buildings.

Lastly, the decomposition scattering powers, i.e., $P_{s}$, $P_{d}$, $P_{v}$, $P_{c}$, and $P_{w}$ can be obtained with the manner similar to the original MCSM decomposition method [12], which will not be further discussed in this paper. We give the mathematical expressions, respectively, below.

(18) $$P_{w}=f_{w}\left({\left|\gamma \right|}^{2}+1+2{\left|\rho \right|}^{2}\right),\;P_{c}=f_{c}=2\left|Im\left[T_{23}-f_{w}\rho^{*}\left(\gamma -1\right)\right]\right|\;P_{v}=f_{v}=\{\begin{array}{c} \left(T_{33}-\frac{1}{2}f_{c}-2{\left|\rho \right|}^{2}f_{w}\right)/\frac{1}{3},\;\mathrm{vegetation}\;\mathrm{area}\\ \left(T_{33}-\frac{1}{2}f_{c}-2{\left|\rho \right|}^{2}f_{w}\right)/\left(\frac{1}{2}+\frac{1}{30}\cos\left(4\theta_{\mathrm{dom}}\right)\right),\;\mathrm{oriented}\;\mathrm{buildings} \end{array}\;P_{d}=f_{d}\left(1+{\left|\alpha \right|}^{2}\right)\;f_{d}=\{\begin{array}{c} T_{22}-\frac{1}{3}f_{v}-\frac{1}{2}f_{c}-\frac{1}{2}\left({\left|\gamma \right|}^{2}-\gamma^{*}-\gamma +1\right)f_{w},\;\mathrm{vegetation}\;\mathrm{area}\\ T_{22}-\left(\frac{1}{2}-\frac{1}{30}\cos\left(4\theta_{\mathrm{dom}}\right)\right)f_{v}-\frac{1}{2}f_{c}-\frac{1}{2}\left({\left|\gamma \right|}^{2}-\gamma^{*}-\gamma +1\right)f_{w},\;\mathrm{oriented}\;\mathrm{buildings} \end{array}\;P_{s}=f_{s}\left(1+{\left|\beta \right|}^{2}\right),\;f_{s}=\{\begin{array}{c} T_{11}-\frac{1}{3}f_{v}-\frac{1}{2}\left({\left|\gamma \right|}^{2}+\gamma^{*}+\gamma +1\right)f_{w},\;\mathrm{vegetation}\;\mathrm{area}\\ T_{11}-\frac{1}{2}\left({\left|\gamma \right|}^{2}+\gamma^{*}+\gamma +1\right)f_{w},\;\mathrm{oriented}\;\mathrm{buildings} \end{array}$$

## 3. Superpixel Generation and Feature Extraction

**Figure 3:**
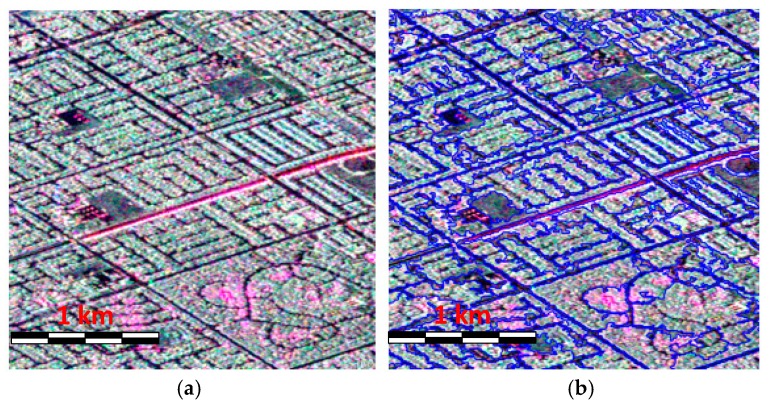
Illustration of the superpixel generation using AIRSAR C band data: (**a**) PolSAR image with Pauli color-coding (red: |HH−VV|; green: |HV|; blue: |HH+VV|), and (**b**) superpixel map.

Object based segmentation and classification for PolSAR images have attracted more and more attention for the reason that it is computational efficient and can reduce the effect of speckle noise by taking the image objects as the processing unit instead of using isolated pixels [1,26]. A superpixel is defined as a local homogeneous region that can preserve most of the object information and adhere to the object boundaries. Furthermore, the features extracted from a superpixel usually exhibit more useful information than those extracted with a pixel since the superpixel may contain many neighborhood pixels. Therefore, it is helpful in improving the image classification accuracy [25]. Several superpixel generation methods for PolSAR images have been proposed. In this paper, we adopt the adaptive polarimetric SLIC, i.e., Pol-ASLIC approach, which was proposed by Xiang et al. [26] to produce superpixels. This method can generate superpixels with an adaptive shape and compactness according to the image content. Furthermore, the boundary adherence is quite good, which shows potential ability to classify the land covers. The detailed information about the Pol-ASLIC can be found in Reference [26]. Figure 3 gives one illustration of the superpixel generation using AIRSAR C band data where we can find that the urban buildings can be discriminated very well and the superpixel boundary is quite clear and accurate.

Note that the superpixels are obtained based on the polarimetric coherency or covariance matrix. Therefore, the polarimetric information can be fully considered and the superpixel boundary can accurately discriminate the objects with different scattering mechanisms. Consequently, in this work we apply the superpixel boundaries on five decomposition scattering power images and extract some texture and spatial features from these images for each superpixel. These features contain: (1) spatial features, i.e., mean and variance values of each superpixel; (2) texture features that include the homogeneity of gray-level co-occurrence matrix (GLCM), GLCM contrast, GLCM dissimilarity, and GLCM entropy of four directions (0°, 45°, 90°, 135°). Therefore, for each superpixel, we can get five polarimetric scattering powers and 90 texture and spatial features. Even though the dimension of this feature set is large, it is much smaller than that of the features used in Reference [21]. It is worth pointing out that, although the dimension of polarimetric scattering power features is dramatically reduced, the polarimetric information of the whole feature set is enough for further classification since all of the spatial and texture features are calculated based on the scattering powers with the assistance of superpixels, which are also obtained based on the scattering matrix. The effectiveness of classification features using in our proposed method is discussed in the following sections.

## 4. Dimensional Reduction of the Features for PolSAR Image Classification

As we discussed in the above section, features with a large dimension may result in information redundancies, which can reduce the classification accuracy. Therefore, feature dimensionality reduction is necessary for image classification and has been widely studied. There are two kinds of dimensionality reduction techniques, i.e., linear and nonlinear methods. In our research, since the features lie on a complicated nonlinear manifold, nonlinear methods are more reasonable than linear dimensionality reduction to discover the intrinsic structure in the data. For PolSAR image classification, we aim to aggregate the pixels of the same class and separating the pixels of different classes. That means the local structure of data needs to be retained so that data pointing in the same class are clustered while data points in different classes are kept away from each other. Therefore, local nonlinear dimensionality reduction techniques are optimal in PolSAR image classification. Now there are many local nonlinear dimensionality reduction techniques such as locally linear embedding (LLE) [29], Laplacian eigenmaps (LE) [31], and local tangent space alignment (LTSA) [47]. These methods are all unsupervised learning algorithms, which consider no prior information on the original data. Furthermore, some parameters need to be set before mapping such as the neighborhood size. This section presents a supervised LLE (S-LLE) method, which can estimate the neighborhood size adaptively and also takes in the discriminable information of training samples.

Let the data matrix Z with size D×M be the input of S-LLE approach, which includes M columns D dimensional feature vectors. The output of S-LLE approach is a new data matrix Y with size d×M where the dimension of feature vector d≤D in the embedded space. S-LLE is implemented with the following steps.

### 4.1. Estimation of the Adjacency Graph

**Figure 4:**
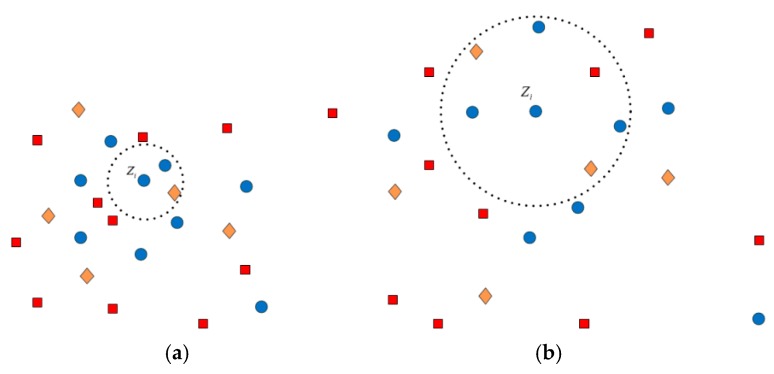
Neighborhood estimation of $Z_{i}$: (**a**) small neighborhood size for aggregated samples; (**b**) large neighborhood size for dispersed samples.

The unsupervised LLE method finds the K nearest neighbors for each data point $Z_{i}$ in the data matrix Z using the Euclidean distance measure and then we can obtain the proximity matrix A with size K×M. The *i*th column contains the indices of K points, which are the neighbors of $Z_{i}$. It can be seen that the neighborhood size K is essential in the LLE algorithm, which should be determined before the feature mapping. In our work, for the sake of exploring geometrical and discrimination information of the data, the neighboring graph can be split into two components, which are the within-class neighboring graph $G_{w}$ and between-class neighboring graph $G_{b}$. Therefore, for each data point $Z_{i}$, we can calculate two neighborhood subsets called $N_{w}\left(Z_{i}\right)$ and $N_{b}\left(Z_{i}\right)$. Note that $N_{w}\left(Z_{i}\right)$ represents the neighbors having the same class label with $Z_{i}$ and $N_{b}\left(Z_{i}\right)$, which denotes the neighbors with different labels with $Z_{i}$. It can be seen that, unlike the classical unsupervised LLE approach, the proposed algorithm adjusts the neighborhood size K, according to the similarity measure between the local sample point $Z_{i}$ and the rest of the samples. The two neighborhood subsets $N_{w}\left(Z_{i}\right)$ and $N_{b}\left(Z_{i}\right)$ are calculated using the equations below.

(19) $$N_{w}\left(Z_{i}\right)=\left\{Z_{j}|L\left(Z_{j}\right)=L\left(Z_{i}\right),ED\left(j,i\right)<D\left(Z_{i}\right)\;\right\}$$

(20) $$N_{b}\left(Z_{i}\right)=\left\{Z_{j}|L\left(Z_{j}\right)\neq L\left(Z_{i}\right),ED\left(j,i\right)<D\left(Z_{i}\right)\;\right\}$$

(21) $$D\left(Z_{i}\right)=\frac{1}{M}\sum_{k=1}^{M}ED\left(k,i\right)$$

where $L\left(Z_{i}\right)$ denotes the class label of $Z_{i}$, ED(k,i) represents the Euclidean distance between data points $Z_{k}$ and $Z_{i}$, and $D\left(Z_{i}\right)$ denotes the average distance between $Z_{i}$ and all other samples. What we can see from Equation (19) is that the set of within-class neighbors of $Z_{i}$, i.e., $N_{w}\left(Z_{i}\right)$, is all data samples with the same class label with $Z_{i}$ and the distance is lower than the average distance associated with $Z_{i}$. There is a similar interpretation for the set of between-class neighbors $N_{b}\left(Z_{i}\right)$. Thus, it is clear that the neighborhood size is adaptive for every data sample, which is shown in Figure 4 and can bypass the setting of parameter K.

### 4.2. Computation of the Weights for Neighbors

Different from the traditional unsupervised LLE approach, in this paper, we divide the single weight matrix W into two sub-weight matrices $W_{w}$ and $W_{b}$, which denote the weights of the within-class neighbor graph and the between-class neighbor graph, respectively. Note that the weight value in the matrices measures the closeness of two data points, which can be further used to measure the contributions of the nearest neighbors to the reconstruction of a given point. The sub-weight matrices $W_{w}$ and $W_{b}$ can be obtained by optimizing the following task as shown below.

(22) $$\{\begin{array}{c} min\overset{M}{\underset{i=1}{\sum }}{\left\|Z_{i}-\overset{M_{1}}{\underset{j=1}{\sum }}W_{w,ij}Z_{j}-\overset{M_{2}}{\underset{j=1}{\sum }}W_{b,ij}Z_{j}\right\|}^{2}\\ W_{w,ij}=W_{b,ij}=0,\;if\;Z_{i}\;and\;Z_{j}\;are\;not\;neighbors\\ \overset{M_{1}}{\underset{j=1}{\sum }}W_{w,ij}+\overset{M_{2}}{\underset{j=1}{\sum }}W_{b,ij}=1 \end{array}$$

where $M_{1}$ and $M_{2}$ are the number of samples within the class neighborhood $N_{w}\left(Z_{i}\right)$ and the between-class neighborhood $N_{b}\left(Z_{i}\right)$, respectively.

### 4.3. Solution of the Mapping Projections

The feature vector mapping projection can be obtained with the optimization of two objective functions as shown below.

(23) $$\{\begin{array}{c} \min\overset{M}{\underset{i=1}{\sum }}{\left\|Y_{i}-\overset{M_{1}}{\underset{j=1}{\sum }}W_{w,ij}Y_{j}\right\|}^{2}\\ \min\overset{M}{\underset{i=1}{\sum }}{\left\|Y_{i}-\overset{M_{2}}{\underset{j=1}{\sum }}W_{b,ij}Y_{j}\right\|}^{2} \end{array}.$$

With this optimization, it can be found that, after the feature mapping, the data points within the same class become closer to each other and data points within different classes are farther away than before. Therefore, after this supervised feature dimensionality reduction, different classes can be distinguished quite well. With the condition $Y^{T}Y=I$, the two objective functions (23) can be further combined into one objective function, which is shown below.

(24) $$\max\left\{\gamma \mathrm{Tr}\left(Y^{T}W_{b}Y\right)+\left(1-\gamma \right)\mathrm{Tr}\left(Y^{T}W_{w}Y\right)\right\}$$

where Tr(·) is the matrix trace operator and the parameter γ is a balance factor that control the within-class and between-class objective function. With $B=\gamma W_{b}+\left(1-\gamma \right)W_{w}$, Equation (24) can be further written as $\mathrm{maxTr}\left(Y^{T}BY\right)$. Then solving this maximization problem is equivalent to optimizing the eigenvector problem BY=λY with the largest nonzero Eigen value.

**Figure 5:**
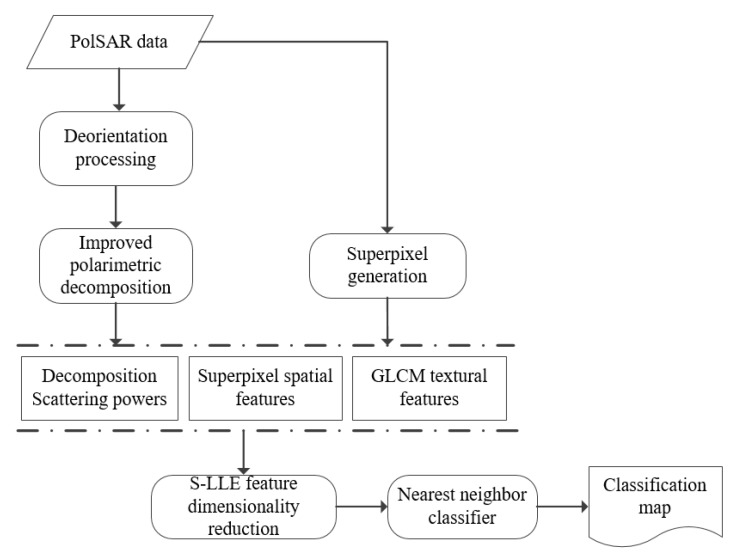
The whole diagram of the proposed methodology.

After mapping the polarimetric, spatial, and textural features from a high dimensional vector to a low dimensional vector, the nearest neighbor (NN) classifier is utilized to achieve the image classification, which adopts the low dimensional feature vector as the input. Figure 5 gives the whole flowchart of our proposed methodology.

## 5. Experimental Results and Discussions

In this section, to test the performance of the proposed image classification method, we carry out experiments on PolSAR dataset from AIRSAR with the C-band. The result of the improved multiple-component model-based decomposition technique is compared with those of other algorithms. In addition, the performance of the S-LLE dimensional reduction method is demonstrated on polarimetric features. Moreover, additional comparisons are also made to investigate the detailed contribution of three components of our method to the LULC classification.

### 5.1. Study Area Description of AIRSAR Data with the C-Band

**Figure 6:**
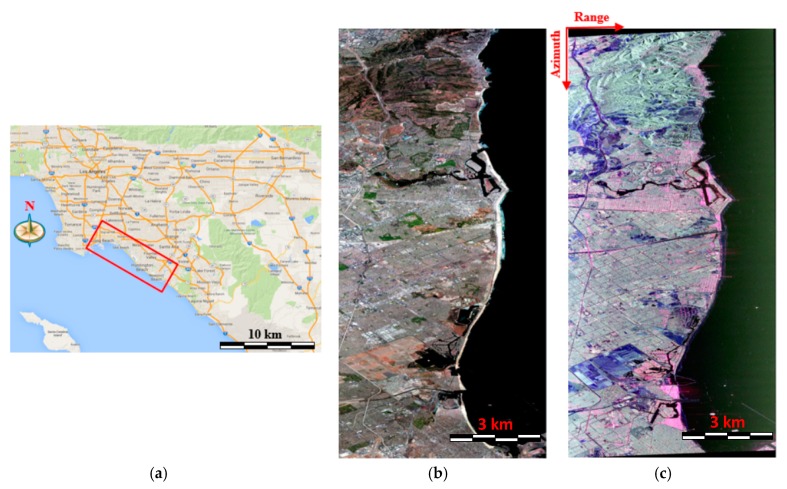
The optical image and PolSAR data of the study area: (**a**) map of the study area; (**b**) optical image that has been registered from Google Earth; (**c**) AIRSAR C band Pauli coded SAR image (red: |HH-VV|; green: |HV|; blue: |HH+VV|).

In this section, we introduce the study area of this paper and the corresponding PolSAR dataset. Figure 6a shows the location site of the study area, which has a coverage of the Long Beach, i.e., a city in Los Angeles County of the USA. Figure 6b presents the high-resolution optical image of the study area, which was obtained from Google Earth. There are some forests, water, bare soil and dense buildings with different orientation angles in this study area. Figure 6c gives the Airborne Synthetic Aperture Radar (AIRSAR) C band PolSAR data with Pauli color coding of this study area where the double-bounce scattering is depicted with a red channel. The volume scattering is described with a green channel and the surface scattering is described with a blue channel. This AIRSAR data were acquired on October 24th in 1998 over the study area. Note that the image rows denote the azimuth direction and the columns correspond to the range direction. The image resolution of the PolSAR data is 4.62 m in the range direction and 3.33 m in the azimuth direction and the image size of is 5291 by 2560 pixels.

From Figure 6b, we can see that the urban areas consist of buildings parallel to the radar flight path and oriented buildings, which are not parallel to the radar flight path. Some buildings are surrounded with vegetation, which creates the back scattering complex. Although the oriented buildings are quite different from the vegetated areas in Figure 6b, they all show a green color in Figure 6c, which means that, similar to vegetation, oriented buildings also contribute to the volume scattering and are often difficult to distinguish from the vegetated areas.

### 5.2. Illustration of the Decomposition Results

**Figure 7:**
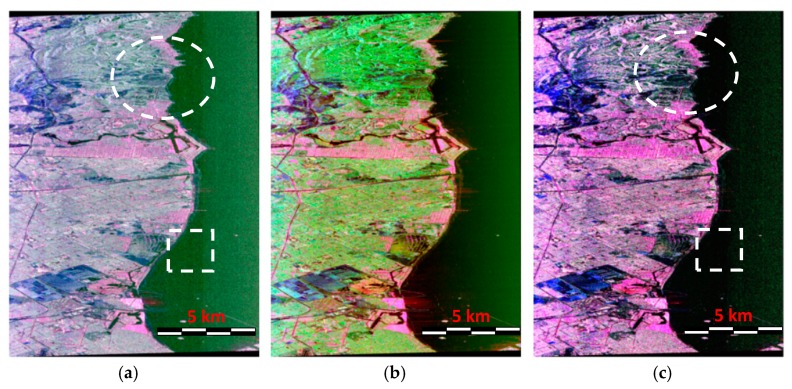
Polarimetric decomposition results of the AIRSAR dataset by (**a**) the original MCSM method, (**b**) the Yamaguchi four-component method with rotated coherency matrix, and (**c**) our proposed improved MCSM decomposition method (red: $P_{d}$; green: $P_{v}$; blue: $P_{s}$).

Figure 7 presents the polarimetric decomposition results of the AIRSAR C band data by different methods, respectively, where Figure 7a gives the results of the original multiple-component model-based decomposition [12]. Figure 7b is the result of the Yamaguchi four-component decomposition with a rotated coherency matrix [11], i.e., Y4R and Figure 7c is the result of our improved multiple-component model-based decomposition. From Figure 7a,b, we can see that, compared with the Y4R method, the MCSM can obtain stronger double-bounce scattering for urban areas especially for the oriented buildings. The reason is that the MCSM decomposition considers wire scattering, which is suitable for the description of the urban scattering mechanism. Therefore, the double-bounce scattering of urban buildings is enhanced and the volume scattering is reduced. However, we can also observe that, for the vegetated areas, the MCSM decomposition performs inadequately because the volume scattering of vegetated areas in the MCSM decomposition is not as strong as with the volume of the Y4R decomposition, which indicates that the volume scattering is suppressed. In comparison, Figure 7a–c shows more satisfactory decomposition as a result. It can be observed that the double-bounce scattering powers of buildings with different orientation angles are strong, which indicate that the HV scattering model of oriented buildings is effective. Moreover, the vegetated area in Figure 7c exhibits stronger volume scattering than that in Figure 7a, which is shown in the circular and rectangular regions marked with white lines. This demonstrates that, using the improved MCSM decomposition, volume scattering within vegetated areas can be well preserved. Consequently, using two different scattering models to describe the volume scattering from oriented buildings and vegetation is valid with our method.

**Figure 8:**
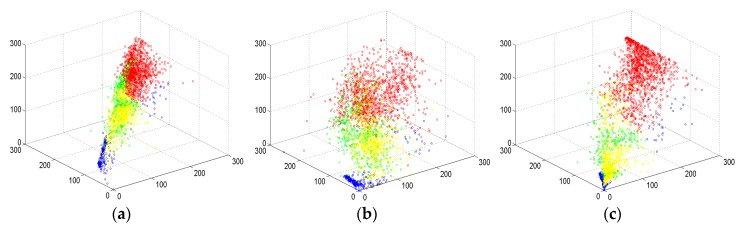
Scattering plots of the decomposition results by (**a**) the MCSM method, (**b**) the Y4R method, and (**c**) the improved MCSM decomposition method (red: urban areas; green: vegetated areas; blue: water areas; yellow: bare soil areas.)

Figure 8 gives the scattering plots of the above three decomposition results. Specifically, the decomposed scattering power images are segmented into many small homogeneous superpixels and each superpixel is associated with a data point in Figure 8. The red color points represent urban areas, green points represent vegetation, yellow color points describe bare soil areas, and blue color points are water. Compared with Figure 8b, what we can see from Figure 8a is that urban areas are more aggregated and fewer vegetation areas are mixed up with urban buildings. However, in the upper-left corner of Figure 8a, there are still some green points in the red color region. In addition, the vegetation points are dispersed and some are mixed up with bare soil areas. In Figure 8c, there are more red points, which indicates that the double-bounce scattering power is enhanced. Moreover, there are very few mixed points between urban and vegetation areas. Bare soil and vegetation can be distinguished better in Figure 8c than in Figure 8a. In forest areas, volume scattering power gets stronger. Water and bare soil points, which are both surface scattering, do not change dramatically in these three methods. This also exists with the decomposition results in Figure 7.

### 5.3. Demonstration of the Supervised S-LLE Dimensional Reduction Method

**Figure 9:**
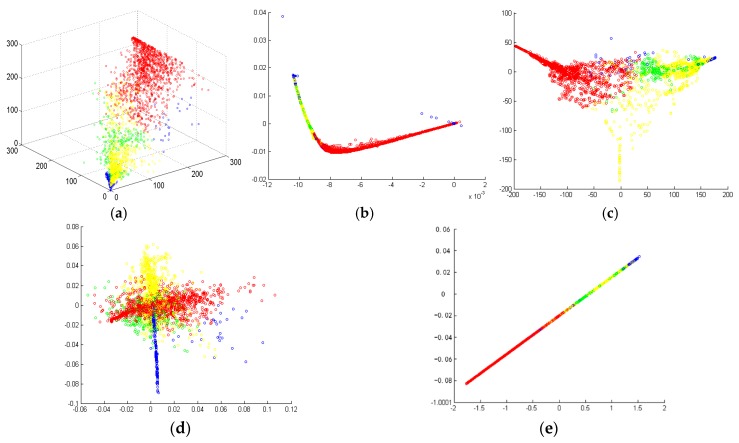
Feature dimensional reduction performance with four different methods: (**a**) original improved MCSM decomposition scattering powers in the 3-D space; (**b**) two-dimensional embedding by the LLE; (**c**) two-dimensional embedding by the LE; (**d**) two-dimensional embedding by the LTSA; (**e**) two-dimensional embedding by the S-LLE (red: urban areas; green: vegetated areas; blue: water areas; yellow: bare soil areas).

**Figure 10:**
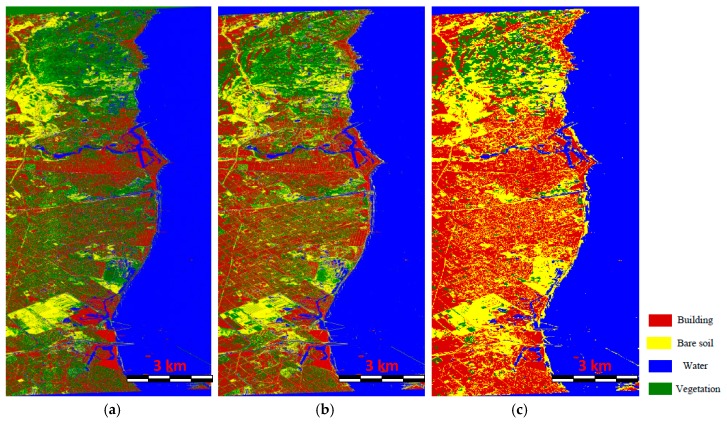
Classification results of the AIRSAR image: (**a**) result of the MCSM-SVM method; (**b**) result of the LE-IF KNN method; (**c**) result of our proposed method.

Since the scattering powers are obtained with the improved decomposition approach in this paper, we demonstrate the effectiveness of the supervised S-LLE on feature dimensional reduction. For the simplicity of observation, only decomposition scattering powers of the improved MCSM technique, which is shown in Figure 9c, are used to compare the performance of various dimensional reduction methods, which can be seen in Figure 10.

From Figure 9, we can see that all the four methods can map high dimensional feature space into low dimensional feature space. However, the degrees of feature distinguishability are quite different. In Figure 9b, it can be seen that, by using the LLE method, urban areas can be easily separated from other classes while bare soil and vegetation are mixed up to a great extent. Water areas are also quite dispersed. Figure 9c,d depict the performance of the LE and LTSA methods, respectively. We can find that the LE can distinguish different classes well even though there are some mixed data points between the water and urban areas. LTSA performs worse than LE because there are lots of mixed points especially in the urban, vegetation, and bare soil areas. In Figure 9c,d, the feature points are all dispersed, which is not beneficial for image classification. In Figure 9e, S-LLE can separate different classes quite well. Furthermore, the feature points aggregate, which is good for the image classification. The above dimensional reduction results are in accordance with Section 4. Thus, it is reasonable using the S-LLE approach to reduce feature redundancy for PolSAR image classification.

### 5.4. Comparison of the Classification Results Using Different Methods

In this subsection, we give the classification result of the K-nearest neighbors classifier with our proposed features and the S-LLE dimensional reduction approach. Moreover, we adopt the original MCSM classification with the support vector machine classifier (MCSM-SVM) [22] and LE-IF with the k-nearest neighbors method (LE-IF KNN) [10] for comparison. In the MCSM-SVM classification, the polarimetric scattering powers obtained from the original MCSM decomposition and the texture features obtained with the GLCM method are regarded as the input of the LIBSVM classifier. Note that the radial basis function kernel is chosen in the LIBSVM and the cost and gamma parameters are selected by cross validation. In the LE-IF KNN method, the Laplacian eigenmaps dimensional reduction method is utilized to map the various polarimetric features into three dimensional features, which are considered as the input of the KNN classifier. In our proposed method, the S-LLE dimensional reduction method is used to map the improved MCSM polarimetric decomposition scattering powers including spatial and textural features extracted from the superpixels into low dimensional features, which are also regarded as the input of the KNN classifier. It is worth pointing out that, in the MCSM-SVM classification, there is no feature dimensional reduction stage while, in the LE-IF KNN classification, the whole feature set is mapped to a three-dimensional vector. Therefore, in order to achieve a fair comparison, we also map the 95-dimensional features to a three dimensional feature vector in our proposed method. The classification results of three methods are shown in Figure 10.

From Figure 10a, it can be found that the MCSM-SVM method can accurately distinguish the vegetation, bare soil, water, and buildings parallel to the radar flight path. However, most of the buildings not parallel to the radar flight path are misclassified to the vegetation. The reason is that, in the MCSM decomposition, although the wire scattering can improve the urban scattering, the double-bounce scattering of the oriented buildings is still seriously underestimated. The HV scattering caused by oriented buildings is regarded as the volume scatting, which leads to scattering mechanism ambiguity. In Figure 10b, we can find that the classification result is much better than that in Figure 10a. The vegetation, bare soil, and water can be well classified. Moreover, most of the buildings including some of the oriented buildings can be effectively discriminated. This is mainly beneficial from various types of polarimetric features, which can provide sufficient information for land cover classification. Moreover, it also can be seen that the LE dimensional reduction technique has the ability to reduce the dimensions of original PolSAR features and preserve the local property at the same time, which is useful for image classification. Nevertheless, there still exist some misclassifications in Figure 10b. For example, lots of roads between the oriented buildings are classified as vegetated areas. What we can observe from Figure 10c is that, not only most of the vegetation, water, and bare soil areas can be correctly classified but also the building with different orientations can be clearly discriminated, which leads to a much better classification result than the other two methods. Although there are some tiny misclassifications between the vegetation and bare soils, most of the main land covers can be correctly classified. More importantly, the classification result of our method has few isolated pixels and noise contamination due to the superpixel-based processing. In addition, the time cost is much lower than those of the other two methods. The satisfactory classification result also demonstrates the effectiveness of the S-LLE dimensional reduction method where the redundant information of different features is reduced and the discriminating information is well preserved. The individual contributions of decomposition, superpixel-based processing, and S-LLE dimensional reduction are discussed in detail in the next subsection.

For the sake of quantitative comparison, we give the confusion matrices of the classification results for different methods in Table 1, Table 2 and Table 3, respectively, where the matrix columns denote the results of the classifiers and the rows represent the true land covers. Considering that the PolSAR data were acquired on 24 October 1998, the ground truth image of the study area is hard to obtain due to the rapid urbanization. Therefore, in this paper, we investigate the optical image with the assistance of Google Earth for acquiring the ground truth. In the calculation of image classification accuracy, we choose samples of different classes from the optical image and the classification image, respectively. It can be seen from Table 3 that the overall accuracy of our proposed method is above 88% and the kappa coefficient is about 0.85, which are quite good. Table 1 and Table 2 give the accuracy assessment measures for the MCSM-SVM classifier and the LE-IF KNN classifier, respectively. We can observe that many buildings are misclassified as vegetation and the bare soils are classified as water and vegetation. Even though the latter misclassification may also exist in our proposed method, large percentage of bare soils reaching 89.32% can still be classified correctly. The overall accuracies of these two classifiers are lower at about 13% and 7% than the percentages of our proposed method. It is worth pointing out that the vegetation accuracy of our proposed method is worse than those of the MCSM-SVM and LE-IF KNN classification methods. The reason is that, after the deterioration processing of the coherency matrix, the double-bounce scattering of vegetation areas may be enhanced to some extent because some trees can exhibit ground-trunk double-bounce and triple-bounce reflections. Moreover, the branch condition may regard this kind of vegetation areas as oriented buildings, which makes the vegetation areas exhibit low volume scattering and high double-bounce scattering. Therefore, there exist some misclassifications in our method.

### 5.5. Contribution Analysis of Three Components to the LULC Classification

**Figure 11:**
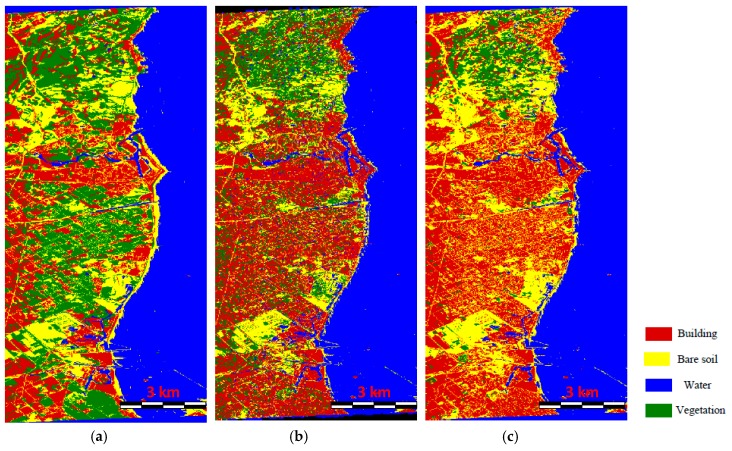
Classification results of the AIRSAR image with a different strategy: (**a**) result without the improved MCSM decomposition; (**b**) result without the superpixel-based processing strategy; (**c**) result without the feature dimensional reduction.

Since this proposed method is conducted by three components, which are the improved MCSM decomposition, the superpixel-based image processing strategy, and the S-LLE dimensional reduction technique, we need to discuss the contribution of each component to the final LULC classification individually. To achieve this task, we carry out three classification experiments. The first is superpixel-based image classification using the Pauli decomposition and the S-LLE dimensional reduction technique, which is designed to test the performance of our improved MCSM decomposition for the classification. To demonstrate the advantage of the superpixel-based processing strategy, the second experiment is pixel-based image classification using the improved MCSM decomposition and the S-LLE dimensional reduction technique. The last experiment is a superpixel-based image classification system using the improved MCSM decomposition powers as well as spatial and textural features without any dimensional reduction method. Figure 11 shows the classification results of the above three experiments.

Figure 11a shows the classification result using the Pauli decomposition from which we can see that lots of building areas are misclassified as vegetation. The reason is that building areas with different polarimetric orientation angles cannot be detected well by the Pauli decomposition features. However, in our improved MCSM decomposition, the oriented buildings and vegetated areas can be distinguished clearly, which is shown in Figure 10c. Moreover, the improved MCSM polarimetric parameters are also beneficial in discriminating the vegetation from the bare soil areas. The pixel-based classification result is depicted in Figure 11b. It can be seen that there are lots of isolated pixels and misclassifications. This is because the speckle noise affects the classification results significantly. Besides the speckle noise within the coherency matrices, there is also large noise in the extracted decomposition parameters. Another problem of Figure 11b is that the time cost of pixel-based classification is really high since there are about $1.3\times 10^{7}$ pixels that need to be processed. However, in our superpixel-based processing approach, there are only about 90,000 superpixels, which reduce the computation load dramatically. Therefore, superpixel-based image analysis contributes substantially to the final accuracy of PolSAR LULC classification. Apart from providing useful textural information to support the classification, other significant advantages are the reduction of the speckle noise effect in PolSAR images and the efficient classification. Figure 11c describes the classification result using the original features without the dimensional reduction where we can see that the result is very similar to that shown in Figure 10c, which demonstrates that the S-LLE can reduce the dimensions of the original feature space while preserving the discriminating information, which is beneficial to the classification. Another advantage of dimensional reduction is that the classification can be increased using low dimensional features.

Table 4 presents the accuracies of the individual classification results shown in Figure 11. It can be seen that, without the improved model-based decomposition scattering powers, the classification accuracy of buildings decreases significantly, which leads to a quite low overall classification accuracy. If the classification is performed with decomposition scattering powers but without the superpixel generation, the classification accuracy of buildings achieves 65.85%, which is much higher than the classification result without decomposition scattering powers. However, the overall classification accuracy is still not satisfactory. Feature dimensional reduction does not have significant impact on the overall classification accuracy. We can see from Table 4 that the classification accuracy is 86.49%, which is a little bit lower than the accuracy of our proposed classification result. This indicates that the feature redundancy may decrease the overall classification accuracy slightly.

**Figure 12:**
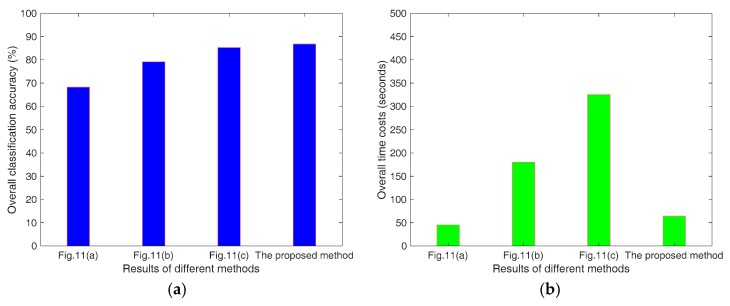
Overall classification accuracy (**a**) and time costs (**b**) of three experiments in Figure 11.

Figure 12 gives the bar graph of overall classification accuracies and time costs of three above experiments. From Figure 12a, we can see that there is a big gap (about 15%) between Figure 11a and the proposed method, which indicates the significant contribution of the improved MCSM decomposition to the LULC classification. The gap between Figure 11b and the proposed method is lower, which is about 7%. This shows that the superpixel-based processing strategy is also important for classification. The S-LLE dimensional reduction technique improves the overall classification accuracy. However, based on Figure 12b, we find that it can reduce the time consuming dramatically, which is from 325.45 s to 64.15 s. Compared to the time costs of Figure 11b with the proposed method, it is also apparent that the superpixel-based processing strategy can also speed up the classification. Our experiments are conducted on a desktop PC with an Intel Core i7-4702 CPU of 2.2 GHz and 8 GB Memory.

## 6. Conclusions

This paper proposed a new approach for PolSAR image classification, which incorporated the improved multiple-component model-based decomposition, superpixel-based image analysis, and the S-LLE dimensional reduction technique. The comparisons between the proposed method and the original MCSM-SVM as well as recently published LE-IF PolSAR classification methods were conducted to demonstrate the performance of LULC classification. The experimental results show that the proposed method can dramatically improve the overall classification accuracy and kappa coefficient. In addition, the processing speed is increased. The detailed discussions of individual contributions of three components indicate that the improved MCSM decomposition parameters are related to the scattering properties of the observed objects. Therefore, they have significant implications for the classification of PolSAR data. The overall accuracy of LULC classification can be improved by 15% if the improved decomposition parameters are used in the classification. The superpixel-based image analysis strategy is quite helpful in improving the accuracy of PolSAR image classification since it can reduce the effect of speckle within PolSAR images and can extract more textural information for the classification than the pixel-based strategy. The overall accuracy of superpixel-based classification of PolSAR data increased by 7% compared with that of conventional pixel-based classification. Furthermore, the time cost of classification is also reduced. The S-LLE dimensional reduction technique contributes to classification efficiency. The similar overall accuracies of the proposed method with and without dimensional reduction methods show that S-LLE can effectively reduce the feature redundancy and, at the same time, can preserve the distinguishability of features.

Future research will focus on the physical interpretations of the low dimensional features obtained by the S-LLE. In addition, more textural features should be investigated to improve the classification accuracy of natural areas.

## Figures and Tables

**Table 1:** Confusion matric of the MCSM-SVM classification result.

Class	Building	Bare Soil	Water	Vegetation	Prod. Acc.
Building	47.33%	1.24%	0.00%	51.43%	47.33%
Bare soil	2.17%	67.55%	10.41%	19.87%	67.55%
Water	0.00%	10.77%	89.12%	0.11%	89.12%
Vegetation	0.28%	2.33%	0.12%	97.27%	97.27%
User. Acc.	95.08%	82.49%	89.43%	57.66%	
Overall accuracy = 75.32%, Kappa coefficient = 0.6709					

**Table 2:** Confusion matric of the LE-IF KNN classification result.

Class	Building	Bare Soil	Water	Vegetation	Prod. Acc.
Building	70.51%	0.77%	0.00%	28.72%	70.51%
Bare soil	1.25%	73.24%	11.78%	13.73%	73.24%
Water	0.00%	9.84%	90.08%	0.08%	90.08%
Vegetation	5.23%	1.01%	0.04%	93.72%	93.72%
User. Acc.	91.58%	86.30%	88.40%	68.78%	
Overall accuracy = 81.88%, Kappa coefficient = 0.7585					

**Table 3:** Confusion matric of the proposed classification result.

Class	Building	Bare Soil	Water	Vegetation	Prod. Acc.
Building	88.67%	1.15%	0.00%	10.18%	88.67%
Bare soil	2.55%	89.32%	1.67%	6.46%	89.32%
Water	0.00%	13.25%	86.57%	0.18%	86.57%
Vegetation	7.32%	1.68%	0.17%	90.83%	90.83%
User. Acc.	89.98%	84.74%	97.91%	84.37%	
Overall accuracy = 88.85%, Kappa coefficient = 0.8513					

**Table 4:** Accuracy of the individual classification results in Figure 11.

	Without Decomposition	Without SuperPixel Processing	Without Feature Dimensional Reduction
Building	32.32%	65.85%	84.66%
Bare soil	80.41%	75.38%	90.01%
Water	82.25%	83.31%	83.25%
Vegetation	82.62%	84.58%	88.07%
Overall	69.40%	77.28%	86.49%
